# Neurocognitive Decline Following Radiotherapy: Mechanisms and Therapeutic Implications

**DOI:** 10.3390/cancers12010146

**Published:** 2020-01-08

**Authors:** Simonetta Pazzaglia, Giovanni Briganti, Mariateresa Mancuso, Anna Saran

**Affiliations:** 1 Laboratory of Biomedical Technologies, ENEA CR-Casaccia, Via Anguillarese 301, 00123 Rome, Italy; simonetta.pazzaglia@enea.it; 2 Department of Radiation Physics Guglielmo Marconi University, Via Plinio 44, 00193 Rome, Italy; giovanni.briganti@unimarconi.it

**Keywords:** neural stem cells, neurogenesis, ionizing radiation, neurocognitive effects

## Abstract

The brain undergoes ionizing radiation (IR) exposure in many clinical situations, particularly during radiotherapy for malignant brain tumors. Cranial radiation therapy is related with the hazard of long-term neurocognitive decline. The detrimental ionizing radiation effects on the brain closely correlate with age at treatment, and younger age associates with harsher deficiencies. Radiation has been shown to induce damage in several cell populations of the mouse brain. Indeed, brain exposure causes a dysfunction of the neurogenic niche due to alterations in the neuronal and supporting cell progenitor signaling environment, particularly in the hippocampus—a region of the brain critical to memory and cognition. Consequent deficiencies in rates of generation of new neurons, neural differentiation and apoptotic cell death, lead to neuronal deterioration and lasting repercussions on neurocognitive functions. Besides neural stem cells, mature neural cells and glial cells are recognized IR targets. We will review the current knowledge about radiation-induced damage in stem cells of the brain and discuss potential treatment interventions and therapy methods to prevent and mitigate radiation related cognitive decline.

## 1. Introduction

Benefit to patients from medical uses of ionizing radiation (IR) has been established beyond doubt. X-ray imaging, including computer tomography (CT) scans and nuclear medicine, is an essential diagnostic instrument for numerous illnesses and has a crucial role in monitoring disease and anticipating prognosis [1]. Moreover, radiation remains, along with surgery and chemotherapy, an essential component of treatment of many types of cancers, with approximately 50% of patients undergoing radiation therapy at some stage during disease [2].

In 2018, the prevalence of central nervous system (CNS) tumors was estimated in 3.5 per 100,000 men and women (all ages) [3]. Chemotherapy for brain tumors is generally restricted by delivery obstacles associated with the blood-brain barrier (BBB) that precludes achieving sufficient concentrations of chemotherapeutic agents in the tumors [4]. Therefore, although several parameters (e.g., cancer site, type and stage) determine choice of the most appropriate therapeutic approach, radiation therapy, beside surgery, remains a main treatment modality for tumors of the CNS and for brain metastases [5,6]. The main objective of radiotherapy is to destroy tumor cells while inflicting the least possible injury to neighboring normal tissues; however, this is often not achievable or feasible [i.e., in case of total-body or whole-brain (WB) irradiation].

**Figure 1:**
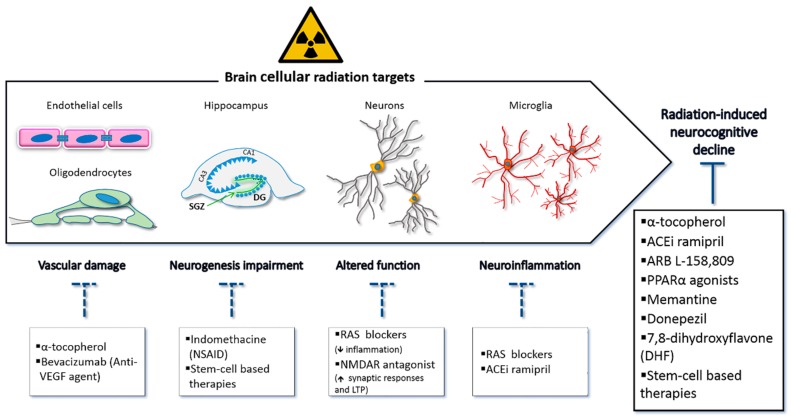
Potential mechanisms triggering radiation-induced cognitive impairment. Brain radiation injury is multifactorial and complex, involving dynamic interactions between multiple cell types. Brain irradiation may cause decline in oligodendrocytes and other glial cells, vascular damage, impaired hippocampal neurogenesis, altered function of adult neurons, and neuroinflammation caused by activated microglia. All these alterations likely contribute to the development of radiation-induced cognitive impairment (upper arrow). Selected strategies to prevent or minimize radiation-induced cognitive dysfunction are shown in the lower boxes, with data derived from both preclinical models and human studies.

Neurocognitive defects are clearly linked with radiation therapy, particularly in children where they represent a major detrimental side effect of life-saving procedures [7]. Cognitive decline may become manifest numerous months to years after irradiation and get progressively worse [8]. With improvement of technologies (e.g., intensity modulated radiotherapy (IMRT), stereotactic radiosurgery, intracranial brachytherapy and limited fraction size) normal tissue damage can be mitigated [2]. However, neurocognitive deficits, including learning, memory, spatial processing, and dementia still persist [3]. Accumulating evidence in animal models suggests that radiation-induced cognitive decline involves damage in multiple neural cell types, causing structural and functional alterations in the brain blood vessels and in glial cell populations, reducing neurogenesis in the hippocampus, altering neuronal function, and increasing neuroinflammation [9] (Figure 1). Overall, brain radiation injury leads to a persistent alteration in the brain’s milieu, with inflammation playing a crucial role [10,11]. Therefore, identification of early treatments with potential to ameliorate or prevent IR-induced CNS damage would be highly beneficial for cancer therapy outcomes [9,12].

In this brief review, we will not be able to cover all topics of interest; rather, we have chosen to focus our analysis on what additional data is needed to improve our understanding of the mechanisms of human radiation-induced cognitive defects, particularly from the standpoint of altered neurogenesis, and on potential strategies that may prevent degenerative processes and their progression to long-lasting or permanent cognitive disability.

## 2. Neural Stem Cells

In spite of the relevance of IR-induced cognitive decline, a serious condition worsening over time, the pathophysiology underlying the progression of this disorder remains scarcely understood, and, despite efforts, truly effective preventive measures or ameliorating treatments are not yet available. IR-induced reduction of brain stem/precursor cells, especially in the subgranular zone (SGZ) of the hippocampus dentate gyrus, is thought to be responsible for decline in hippocampus-related functions, i.e., learning, memory, and processing of spatial information [13]. IR-induced deficits in processes underlying these key functions in animal models are coupled with increased apoptotic processes in the hippocampus [14]. Similarly, substantial and protracted stem cell reduction occurs in the subventricular zone (SVZ) of the anterior lateral ventricles in a dose-dependent manner [15]. IR can also deeply impact adult neurogenesis, mainly by preventing mitosis and integration of new neurons into the circuitry of these critical regions [16,17,18], with long-lasting related sequelae for memory and learning. This, at least in rodent models, is a fairly well consolidated picture.

The complexity of the progressive cognitive disability due to IR brain exposure cannot be fully explained by alteration of a single cell type, and the pathogenesis of radiation-induced cognitive injury is likely dependent on dynamic connections between multiple cell types (i.e., neurons, microglia and astrocytes). The local microenvironment is increasingly being implicated in the functionality of these cell types, which is orchestrated by a variety of crucial factors, comprising oxygen supply, nutritional status, hormonal and trophic influences, but also through the cellular and humoral pathways of the immune system [19,20]. Brain irradiation triggers a process that leads to uncontrolled activation of microglia and substantial presence of macrophage-secreted cytokines. Studies in hippocampal and cortical regions isolated from irradiated rat brains showed significantly upregulated expression of inflammation markers interleukin 6 (IL-6), interleukin-1β (IL-1β) and tumor necrosis factor-α (TNF-α) [21]. Neuronal turnover can be prevented by a hostile microenvironment, for example that fostered by chronic inflammatory response, and by apoptosis of neural cells. As a secondary result of apoptotic death of microvascular endothelial cells, severe impairment of the BBB has been detected in irradiated rats [22]. The BBB is made by the brain microvasculature that, due to peculiar anatomic and physiologic features such as the intercellular tight junctions, selectively restricts the BBB paracellular diffusion of compounds. Many systemic disorders are characterized by disruption of the BBB, whereby plasma components, immune molecules or cells may enter the brain and consequently activate resident microglia [23] resulting in a significant gap between hippocampus stem cells and their microvascular supply, increased neuronal apoptotic death and reduced generation of new granule neurons by neural stem cells through dysfunction of the neurogenic niche/niches. In depth understanding of the molecular and cellular mechanisms involved in such effects may provide useful targets for possible pharmaceutical and cellular intervention strategies preventing or improving IR-induced brain injury.

However, it should be pointed out that the anatomical and physiological differences among species represent an important limiting issue in effective translation of fundamental research results from animal studies to humans. Several key points deserve attention.

### 2.1. Human Adult Neurogenesis

In the 1960s, it was first discovered that, similar to other vertebrates, such as fish and amphibians, adult neurogenesis also occurs in mammals: new nervous system cells continue to grow in the brain, even as animals get older. It has since become generally accepted that the hippocampus is a brain region wherein adult generation of new neurons occurs in humans as it does in animals. However, while in many mammalian species hippocampal adult neurogenesis is well established and recognized, evidence in humans is relatively sparse [24], and it is still debated. A report by Sorrells and colleagues in 2018 showed that neurogenesis is absent from the adult human hippocampus [25], in line with previous findings [26,27], and that hippocampal neurogenesis is also rare or absent in adult rhesus macaque. In the same year, a study by Boldrini et al. [28] found that human neurogenesis persists into old age. In 2019, two further articles showed persistent neurogenesis in the hippocampus of aging brains and in patients with mild cognitive dysfunction and Alzheimer’s disease [29,30]. The paper by Sorrells and colleagues raised heated discussions, and the controversy is not likely to be resolved in the near future.

Indeed, assessing adult neurogenesis in humans is challenging. Major evidence originates from studies using incorporation of thymidine analogs in the DNA of dividing cells, and from investigations based merely on immunohistochemistry to detect cell proliferation markers in human brain tissue obtained at autopsy. Studying postmortem brain tissues is hampered by several potential technical obstacles. Indeed, standardization of essential methodological requirements would be needed, e.g., the maximum premortem agonal period, maximum time elapsed from death to tissue fixation and fixation times [31]. Once methods for sample collection are standardized, reconciling different findings on the persistence of neurogenesis in the human adult brain will require more comprehensive analyses, e.g., using single-cell RNA sequencing, overcoming the limitations of antibodies and marker specificity as well as individual variability of marker expression, organizing open data repositories of human neurogenomics, and establishing brain-tissue bank/s, open to researchers, from large patient cohorts [32,33]. Advancements in non-invasive imaging and biomarker studies would also help advance the field.

### 2.2. Radiation Effects on Cognitive Function

A number of epidemiological studies have been carried out to assess the risks for neurocognitive decline or cerebrovascular disease (an important cause of disability and dementia) associated with radiation exposure. The populations studied were mainly atomic-bomb survivors, cancer survivors and occupational cohorts (reviewed in [22]). These studies largely agree with the notion that ongoing/incomplete developmental processes in prenatal age or in childhood underlie the human brain vulnerability to irradiation during younger ages. Moreover, the increased time for damage to be expressed when exposure occurs at young ages increases the lifetime risks of developing IR-induced long-term neurocognitive effects. In adults there is less compelling evidence, especially from atomic-bomb survivors or occupational cohorts. Notably, the multimodal therapy protocols, mostly adopted for brain cancers, hamper the interpretation of data on IR-induced cognitive dysfunction coming from studies on cancer survivors [34,35,36,37,38,39] due to the arduous distinction between the damaging effects of radiotherapy from those dependent on primary tumor, surgical procedures, and chemotherapy [22].

Besides rodents, radiation effects on the brain have also been studied using healthy non-human primates without chemotherapy or surgery as confounding factors. Robbins et al., using adult male rhesus monkeys in a head irradiation study with 40 Gy in 5-Gy biweekly fractions for 4 weeks [40], reported a substantial reduction in cognitive function. The rhesus monkeys showed pathology similar to humans exposed to radiation and comparable cognitive decline. This followed a temporal pattern similar to the cognitive sequelae of human intracranial radiotherapy patients, with early decline at about one month from exposure, followed by temporary recovery in the next one‒two months, and progressive decline of performance through 11 months after irradiation. Neuropathologic changes that could have served as the basis for those cognitive effects were published in a subsequent study [41] and included severe multifocal necrosis of the forebrain, midbrain and brainstem. Early CNS damage eventually preceding the cognitive deterioration observed in rhesus monkeys was not analyze; therefore, it is not known which structure/cell type, if any, was initially affected.

A comparative view highlights the importance of using models other than rodents when investigating the biological processes of adult neurogenesis. Non-human primates have strong similarities with humans regarding anatomical, physiological and immunological features; social behavior and cognitive functions also resemble those found in humans. Thus, they represent an important translational model of human disease and a critical bridge between preclinical and clinical research on IR-induced cognitive decline and approaches for its prevention/mitigation.

**Figure 2:**
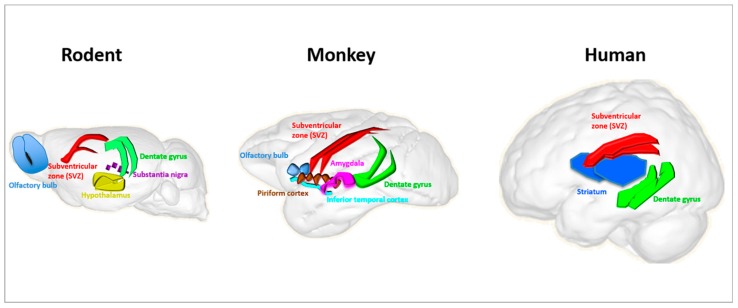
Schematic illustration of reported adult neurogenesis sites in rodent, monkey and human brains. Neurogenesis takes place throughout life in the hippocampal dentate gyrus and the subventricular zone (SVZ) in rodents and is generally accepted to take place in adult monkey and human brains. The output of new neurons from the SVZ to the olfactory bulb is different between humans and other mammals, and humans exhibit very pronounced striatal adult neurogenesis compared to rodents and non-human primates (reviewed in [42]). A different number of neurogenic zones can be detected in adult rodents, monkeys and humans; hypothalamus and substantia nigra in rodents; amygdala, piriform cortex and inferior temporal cortex in monkeys; and striatum in humans. This figure is inspired by the Scalable Brain Atlas website and its 3-D Composer.

Of note, however, whereas adult neurogenesis is preserved among mammals, significant differences remain between rodents, primates, and humans, highlighting how cautious we ought to be in generalizing the results on adult neurogenesis from rodents to non-human primates and, finally, to humans. Differences range from structural to functional. For the scope of this review, it is useful to list only a few, comprising: the different dynamic of neuronal maturation, with much longer times in non-human primates than in rodents, and lack of human data; the different cytoarchitecture of the SVZ in rodents compared to primates; the migration of newborn cells from the SVZ to the olfactory bulb in rodents and monkeys, with sparse evidence for humans; very pronounced striatal adult neurogenesis in humans compared to rodents and non-human primates (reviewed in [42]); finally, a different number of neurogenic zones detectable in adult rodents, monkeys and humans [43] (Figure 2).

## 3. Strategies for Preventive and Therapeutic Measures: Current Knowledge and Perspectives

### 3.1. Improvement of Techniques in Radiation Therapy

External beam radiation therapy is an essential part of treatment of brain tumors and brain metastases [2,3]. Radiation techniques were initially based on 2D radiation therapy by means of rectangular fields built on basic X-ray imaging for field placement verification. WB irradiation has been used for many decades [44] and is still employed for treatment of patients with multiple brain metastases and for prophylactic cranial irradiation of small-cell and non-small-cell lung cancer (SCLC, NSCLC) patients [45]. Although shown to prolong survival, WB radiation therapy (WBRT) may be associated with substantial cognitive impairment [45,46].

Advances in radiotherapy and imaging technology—from 3D conformal radiotherapy based on CT imaging, through IMRT, to more sophisticated techniques—have radically improved delineation of treatment volumes and delivery of highly conformal irradiation, reducing damage to adjoining normal tissue [47]. Previous studies have suggested that techniques allowing selective avoidance of the hippocampal neural stem-cell compartment cause a lower degree of cognitive impairment relative to WBRT [13,48]. With the development of IMRT, volumetric-modulated arc therapy (VMAT) and intensity modulated proton therapy (IMPT), hippocampal-sparing WBRT has been increasingly used as an adjuvant to surgery in the treatment of primary brain neoplasms and in management of brain metastases [49,50,51]. The results of ongoing randomized clinical trials will help to clarify the role of hippocampal-sparing WBRT in cognitive preservation [45,46,52]. They will also potentially open the way for other challenging radiotherapy technique trials.

Stereotactic radiosurgery (SR) allows delivery of highly conformal therapeutic doses to the target by simultaneous exposure to multiple intersecting beams, with minimal radiation to surrounding normal brain [45,46]. SR is increasingly used for patients with limited brain metastases (<5), with or without adjuvant WB radiation therapy, or with systemic therapy [45,53]. The combination of SR and immune checkpoint inhibitors is progressively more used in the treatment of brain metastases, based on reported synergistic effects between SR and immune system modulation [54]. These novel strategies suggest the opportunity to delay or entirely avoid the deleterious neurocognitive effects of WBRT administration.

### 3.2. Pharmacologic Interventions

The permanent cognitive decline that is often associated to brain radiotherapy is likely multifactorial in its origins; thus, improved understanding of the mechanisms of IR-induced cognitive decline will be needed in order to select candidate therapeutics. Figure 1 shows a summary of potential therapeutic measures possibly preventing IR-induced cognitive dysfunction, with indication of the known targeted alterations.

Experiments in rats showed that IL-6-mediated neuroinflammation alone blocks neuronal differentiation of hippocampal NSCs and that administration of indomethacin, a non-steroidal anti-inflammatory drug of common use, is partially effective in restoring neurogenesis after brain irradiation [55]. Significant improvement in global cognition, memory and executive function with use of α-tocopherol, the most abundant and extensively studied form of vitamin E, was observed in a phase II trial of patients with temporal lobe radionecrosis following radiation therapy for nasopharyngeal carcinoma, after administration prolonged for one year [56]. Prevention or reduction of oxidative stress-induced brain damage has been reported following pre- or post-radiation treatment of mice with α-lipoic acid; similarly, treatment of rats with melatonin was able to significantly reduce edema, necrosis, and neuronal degeneration [22]. A major cause of IR-induced tissue damage is the generation of reactive oxygen species. Reduced sensitivity to IR-induced damage in hippocampal-related functions was shown in a study with knockout mice lacking the extracellular antioxidant enzyme superoxide dismutase (EC-SOD KO) [57]. Subsequent interesting work by the same group undertook manipulation of the redox balance in the hippocampus using a bigenic mouse model overexpressing EC-SOD (OE) in the granule cell layer, in an overall EC-SOD-deficient environment. They showed that OE and KO mice exhibit similar hippocampal-related functions following cranial irradiation; molecular examinations suggested that this may be governed by distinct mechanisms, with neurotrophic factors influencing IR responses in OE mice and dendritic maintenance playing an important role in the KO environment [58].

The blockade of the renin-angiotensin system (RAS) can effectively modulate radiation-induced brain injury, presumably through inhibition of renin-angiotensin system-mediated neuroinflammation [59]. In the brain, RAS is involved in modulation of cognition and memory [60]. Blockade of the RAS using either angiotensin converting enzyme inhibitors (ACEi) or angiotensin II receptor blockers (ARB) has been used in cranially-irradiated rats. Chronic administration of the ACEi ramipril starting 24 h postirradiation reduced the deleterious effects of a total-body single dose of 10 Gy on neurogenesis in the rat dentate gyrus but did not prevent neuroinflammatory effects [61]. More recently, ramipril administered before, during, and after fractionated WB irradiation (cumulative dose of 40 Gy) prevented both radiation-induced cognitive impairment and increased microglial activation, despite reduced hippocampal neurogenesis in the context of pharmacologic blockade of angiotensin II-mediated inflammation [62], suggesting that both the radiation dosing scheme and the timing/dose of ramipril administration may modulate the effects of treatment. Finally, while ARB L-158,809 was able to mitigate cognitive dysfunction after rat WB irradiation with a dose of 10 Gy [63], the drug did not improve the neuroinflammatory microglial response or restore hippocampal neurogenesis [64]. The ramipril data strongly suggest that the detrimental effects of IR on cognition could involve alterations in neuronal subsets with higher degree of maturation—in addition to neurogenic niches—that may collectively influence the structural and synaptic plasticity of the IR-exposed CNS [65].

New therapeutic strategies may also evolve from extrapolation of results from other CNS diseases. The peroxisomal proliferator-activated receptors (PPARs) are ligand-activated transcription factors producing anti-inflammatory and neuroprotective effects in several CNS disorders [66]. Administration of PPARα agonist fenofibrate following a WB dose of 10 Gy initially prevented the decrease of hippocampal neurons and inhibited microglial activation in mice [67]. Later on, in a study of fractionated WB irradiation of rats with a higher total dose (40 Gy, two 5 Gy fractions/week for 4 weeks), Greene-Schloesser et al. showed that fenofibrate prevented radiation-induced cognitive deficits but did not mitigate reduced neurogenesis or increase in activated microglia compared with non-drug treated rats [68], highlighting the necessity to look at different brain regions and not the hippocampus alone when investigating IR-induced cognitive dysfunction. Oral administration of the PPARγ agonist pioglitazone, before, during or following fractionated WB irradiation (40 Gy) significantly improved cognitive impairment relative to untreated irradiated rats [69].

Minocycline is a tetracycline antibiotic with inhibitory effects on the brain’s microglia and is currently being investigated as treatment for depression [70]. In preclinical studies, radiation-induced neuronal apoptosis was significantly inhibited in rats (WB single dose of 20 Gy), leading to decreased apoptosis of newborn neurons and improved cognitive performance [71], effects that were activated by enhancement of radiation-induced AMPKα1-mediated autophagy [72]. A recent study in mice with neural-specific deletion of the autophagy related 7 (Atg7) gene, WB irradiated with a single dose of 6 Gy, showed prevention of IR-induced neural stem and progenitor cell death, suggesting autophagy as a possible target to counteract IR-induced neural cell death and related neurocognitive dysfunction [73].

Acetylcholinesterase inhibitors, e.g., donepezil, are being pursued as they have produced some positive results in Alzheimer’s and other dementias [11]. Acetylcholine precursors have also found frequent use for treatment of stroke and many types of dementia. Plangár et al. showed neuroprotective effects of L-alpha-glycerylphosphorylcholine against increased macrophage density, reactive gliosis, calcification and extent of demyelination in an experimental rat model treated with 40-Gy partial-brain irradiation [74].

Recently, small-molecule tropomyosin receptor kinase B (TrkB) agonist 7,8-dihydroxyflavone (DHF) has attracted substantial interest as a new possible option for management of traumatic brain injury [75]. Cognitive impairments following cranial irradiation of mice (5 Gy γ rays) could also be mitigated by treatment with DHF by activating TrkB signaling and downstream survival PI3K/Akt or Erk pathways, thus decreasing neuronal damage [76]. Spatial, contextual, and working memory were significantly rescued by DHF treatment, and the beneficial effects were persistent (i.e., three months after end of treatment).

Memantine is approved in the US and the EU for treatment of mild-to-moderate Alzheimer’s disease. The neuroprotective effects of memantine are attributed to the blockade of N-methyl-D-aspartate receptor, which is involved in learning and memory [77]. A randomized, double-blind, placebo-controlled study revealed that administration of memantine during and after radiation therapy to the whole brain prompted improved cognitive performance, delaying time to cognitive dysfunction and decreasing the rate of decay in memory, executive function, and processing speed [78]. Memantine administration and WB radiation therapy with and without hippocampal sparing are currently being examined in a clinical trial (NRG CC-001) [11].

Selected strategies to prevent or minimize radiation-induced cognitive dysfunction are summarized in the lower boxes of Figure 1.

### 3.3. Stem-Cell Transplantation Approaches

Restoration/increase of neurogenesis by stem cell transplantation is an area of growing interest in the field of neurocognitive decline, representing a promising approach to replenish lost neurons in cases of neurodegeneration or injury. Regarding radio-neuroprotection, it has been shown that transplantation of human neural stem cell mitigates IR-induced cognitive decline in head irradiated mice [79,80]. Matching the results obtained from other CNS disorders (i.e., stroke, Huntington’s, Alzheimer’s and Parkinson’s disease, but also aging), the transplanted neural stem cells are not only capable of differentiating into neurons, but also evolve into different neural cell types, e.g., oligodendrocytes, astrocytes and endothelial cells [81]. Similar neuroprotective effects after head-only irradiation were shown in recent work involving intrahippocampal transplantation of microvesicles secreted from human neural stem cells. The transplanted microvesicles were hypothesized to act through a trophic support mechanism contributing to reduce inflammation and preserve the host neuronal structure. In immunodeficient rats, transplantation of microvesicles to ameliorate cognition, as compared to direct engrafting of human neural stem cells, may circumvent the risk of teratoma formation in the brain and minimize immune rejection, which would then require immunosuppression [82]. Oligodendrocyte progenitors derived from human embryonic stem cells engrafted into the forebrain and cerebellum of young rats were shown to remyelinate the irradiated brain and to ameliorate cognitive deficits, while rescue of motor coordination required concurrent oligodendrocyte progenitor injections into the cerebellum [83]. Soria et al. showed that intranasally delivered human mesenchymal stem cells could promote repair of IR-induced brain damage in mice, improving neurological function and conferring protection against inflammation, oxidative stress, and neuronal loss [84]. Treatments using stem-cell therapies suggest that the IR-induced decrease in neurogenesis can be prevented, but it will take more research before they are successfully translated to the clinic. The safety and the efficacy of mesenchymal stem cell therapy in humans are currently been investigated in seven ongoing clinical trials. Intravenous stem-cell administration has been adopted in the majority of them, as this is a much less invasive procedure than intracranial injections [85]. The completion of these important clinical studies will help address the potential of stem-cell therapy for mitigation of neurocognitive impairment in humans.

## 4. Summary

Although the evidence of the involvement of adult neurogenesis in cognitive processes in rodents is substantial, there is also a significant body of evidence indicating the opposite (reviewed in [42]). For example, performance in the Morris water maze, as well as in other tests of memory and learning was not altered by genetic impairment or ablation of neurogenesis [86,87]. To explain the reason(s) for such discrepancies we should consider that the CNS includes several structures that are possibly sensitive to radiation. Differentiated neurons may not be inert to radiation as previously thought; thus, neuronal dysfunction and not neuronal loss can be, to some extent, the driver of radiation induced cognitive impairment [88,89].

These findings suggest that radiation-induced cognitive decline may be associated with adult neurogenesis to a variable degree, from no connection to being highly dependent on the incorporation of new neurons in the hippocampus or other key neurogenic zones, depending on the status of brain parenchyma, neuroinflammatory conditions, the appropriate choice of behavioral tests, strain variability, time of sampling, and remaining technical problems.

## 5. Conclusions

Brain radiation injury is multifactorial and complex and is characterized by a range of molecular/cellular/tissue alterations. A number of encouraging therapeutic approaches against late effects on the irradiated brain have been developed. However, these have been investigated mostly in rodents, with the notable disadvantage that this model lacks anatomic and physiological similarity to humans [22,41], which limits translation of the findings. A better understanding of the relevance of hippocampus neurogenesis in radiation-induced neurocognitive effects in different species is needed, but this will presumably not be accomplished in the short-term. Elucidation of involved cellular and molecular mechanisms will help development of new preventive and therapeutic methods mitigating the adverse long-term sequelae of brain irradiation. Because several medical disorders with a high accumulative burden of disease, like stroke, HIV infection, traumatic brain injury, and many others, are characterized by altered hallmarks of neuroinflammation, targeting neuroinflammation should remain central in preventing/mitigating radiation-induced cognitive development before new light is shed on the frequently detected dissociation between neurogenesis status and cognitive performance. New therapeutic strategies may also evolve from extrapolation of results from other CNS diseases or from stem-cell approaches. Progress from all these fields may help suggest further possible ways of intervention.

## References

[1] Thomas G.A., Symonds P. (2016). Radiation exposure and health effects—is it time to reassess the real consequences?. Clin. Oncol. (R. Coll. Radiol.).

[2] Baskar R., Lee K.A., Yeo R., Yeoh K.W. (2012). Cancer and radiation therapy: Current advances and future directions. Int. J. Med. Sci..

[3] Michaelidesová A., Konířová J., Bartůněk P., Zíková M. (2019). Effects of radiation therapy on neural stem cells. Genes.

[4] Wang Z., Sun H., Yakisich J.S. (2014). Overcoming the blood-brain barrier for chemotherapy: Limitations, challenges and rising problems. Anticancer Agents Med. Chem..

[5] Delaney G., Jacob S., Featherstone C., Barton M. (2005). The role of radiotherapy in cancer treatment: Estimating optimal utilization from a review of evidence-based clinical guidelines. Cancer.

[6] Chi A., Komaki R. (2010). Treatment of brain metastasis from lung cancer. Cancers.

[7] Askins M.A., Moore B.D. (2008). Preventing neurocognitive late effects in childhood cancer survivors. J. Child Neurol..

[8] Monje M., Dietrich J. (2012). Cognitive side effects of cancer therapy demonstrate a functional role for adult neurogenesis. Behav. Brain Res..

[9] Makale M.T., McDonald C.R., Hattangadi-Gluth J.A., Kesari S. (2017). Mechanisms of radiotherapy-associated cognitive disability in patients with brain tumours. Nat. Rev. Neurol..

[10] Russo I., Barlati S., Bosetti F. (2011). Effects of neuroinflammation on the regenerative capacity of brain stem cells. J. Neurochem..

[11] Jenrow K.A., Brown S.L., Lapanowski K., Naei H., Kolozsvary A., Kim J.H. (2013). Selective inhibition of microglia-mediated neuroinflammation mitigates radiation-induced cognitive impairment. Radiat. Res..

[12] Prise K.M., Saran A. (2011). Concise review: Stem cell effects in radiation risk. Stem Cells.

[13] Gondi V., Tomé W.A., Mehta M.P. (2010). Why avoid the hippocampus? A comprehensive review. Radiother. Oncol..

[14] Yang L., Yang J., Li G., Li Y., Wu R., Cheng J., Tang Y. (2017). Pathophysiological responses in rat and mouse models of radiation-induced brain injury. Mol. Neurobiol..

[15] Tada E., Yang C., Gobbel G.T., Lamborn K.R., Fike J.R. (1999). Long-term impairment of subependymal repopulation following damage by ionizing irradiation. Exp. Neurol..

[16] Mizumatsu S., Monje M.L., Morhardt D.R., Rola R., Palmer T.D., Fike J.R. (2003). Extreme sensitivity of adult neurogenesis to low doses of X-irradiation. Cancer Res..

[17] Casciati A., Dobos K., Antonelli F., Benedek A., Kempf S.J., Bellés M., Balogh A., Tanori M., Heredia L., Atkinson M.J. (2016). Age-related effects of X-ray irradiation on mouse hippocampus. Oncotarget.

[18] Kempf S.J., Casciati A., Buratovic S., Janik D., von Toerne C., Ueffing M., Neff F., Moertl S., Stenerlöw B., Saran A. (2014). The cognitive defects of neonatally irradiated mice are accompanied by changed synaptic plasticity, adult neurogenesis and neuroinflammation. Mol. Neurodegener..

[19] Lumniczky K., Szatmári T., Sáfrány G. (2017). Ionizing radiation-induced immune and inflammatory reactions in the brain. Front. Immunol..

[20] Chesnokova V., Pechnick R.N., Wawrowsky K. (2016). Chronic peripheral inflammation, hippocampal neurogenesis, and behavior. Brain Behav. Immun..

[21] Lee W.H., Sonntag W.E., Mitschelen M., Yan H., Lee Y.W. (2010). Irradiation induces regionally specific alterations in pro-inflammatory environments in rat brain. Int. J. Radiat. Biol..

[22] Hladik D., Tapio S. (2016). Effects of ionizing radiation on the mammalian brain. Mutat. Res..

[23] Benatti C., Blom J.M., Rigillo G., Alboni S., Zizzi F., Torta R., Brunello N., Tascedda F. (2016). Disease-induced neuroinflammation and depression. CNS Neurol. Disord. Drug Targets.

[24] Danzer S.C. (2018). Adult neurogenesis in the human brain: Paradise lost?. Epilepsy Curr..

[25] Sorrells S.F., Paredes M.F., Cebrian-Silla A., Sandoval K., Qi D., Kelley K.W., James D., Mayer S., Chang J., Auguste K.I. (2018). Human hippocampal neurogenesis drops sharply in children to undetectable levels in adults. Nature.

[26] Sanai N., Nguyen T., Ihrie R.A., Mirzadeh Z., Tsai H.H., Wong M., Gupta N., Berger M.S., Huang E., Garcia-Verdugo J.M. (2011). Corridors of migrating neurons in the human brain and their decline during infancy. Nature.

[27] Dennis C.V., Suh L.S., Rodriguez M.L., Kril J.J., Sutherland G.T. (2016). Human adult neurogenesis across the ages: An immunohistochemical study. Neuropathol. Appl. Neurobiol..

[28] Boldrini M., Fulmore C.A., Tartt A.N., Simeon L.R., Pavlova I., Poposka V., Rosoklija G.B., Stankov A., Arango V., Dwork A.J. (2018). Human hippocampal neurogenesis persists throughout aging. Cell Stem Cell.

[29] Tobin M.K., Musaraca K., Disouky A., Shetti A., Bheri A., Honer W.G., Kim N., Dawe R.J., Bennett D.A., Arfanakis K. (2019). Human Hippocampal Neurogenesis Persists in Aged Adults and Alzheimer’s Disease Patients. Cell Stem Cell.

[30] Moreno-Jiménez E.P., Flor-García M., Terreros-Roncal J., Rábano A., Cafini F., Pallas-Bazarra N., Ávila J., Llorens-Martín M. (2019). Adult hippocampal neurogenesis is abundant in neurologically healthy subjects and drops sharply in patients with Alzheimer’s disease. Nat. Med..

[31] Nogueira A.B., Nogueira A.B., Veiga J.C.E., Teixeira M.J. (2018). Letter: Human hippocampal neurogenesis drops sharply in children to undetectable levels in adults. Neurosurgery.

[32] Kempermann G., Gage F.H., Aigner L., Song H., Curtis M.A., Thuret S., Kuhn H.G., Jessberger S., Frankland P.W., Cameron H.A. (2018). Human adult neurogenesis: Evidence and remaining questions. Cell Stem Cell.

[33] Gage F.H. (2019). Adult neurogenesis in mammals. Science.

[34] Mulhern R.K., Merchant T.E., Gajjar A., Reddick W.E., Kun L.E. (2004). Late neurocognitive sequelae in survivors of brain tumours in childhood. Lancet Oncol..

[35] Duffner P.K. (2010). Risk factors for cognitive decline in children treated for brain tumors. Eur. J. Paediatr. Neurol..

[36] Hoang D.H., Pagnier A., Guichardet K., Dubois-Teklali F., Schiff I., Lyard G., Cousin E., Krainik A. (2014). Cognitive disorders in pediatric medulloblastoma: What neuroimaging has to offer. J. Neurosurg. Pediatr..

[37] Crossen J.R., Garwood D., Glatstein E., Neuwelt E.A. (1994). Neurobehavioral sequelae of cranial irradiation in adults: A review of radiation-induced encephalopathy. J. Clin. Oncol..

[38] Surma-aho O., Niemelä M., Vilkki J., Kouri M., Brander A., Salonen O., Paetau A., Kallio M., Pyykkönen J., Jääskeläinen J. (2001). Adverse long-term effects of brain radiotherapy in adult low-grade glioma patients. Neurology.

[39] Meyers C.A., Brown P.D. (2006). Role and relevance of neurocognitive assessment in clinical trials of patients with CNS tumors. J. Clin. Oncol..

[40] Robbins M.E., Bourland J.D., Cline J.M., Wheeler K.T., Deadwyler S.A. (2011). A model for assessing cognitive impairment after fractionated whole-brain irradiation in nonhuman primates. Radiat. Res..

[41] Hanbury D.B., Robbins M.E., Bourland J.D., Wheeler K.T., Peiffer A.M., Mitchell E.L., Daunais J.B., Deadwyler S.A., Cline J.M. (2015). Pathology of fractionated whole-brain irradiation in rhesus monkeys (*Macaca mulatta*). Radiat. Res..

[42] Augusto-Oliveira M., Arrifano G.P.F., Malva J.O., Crespo-Lopez M.E. (2019). Adult hippocampal neurogenesis in different taxonomic groups: Possible functional similarities and striking controversies. Cells.

[43] Lei W., Li W., Ge L., Chen G. (2019). Non-engineered and engineered adult neurogenesis in mammalian brains. Front. Neurosci..

[44] Leibel S.A., Sheline G.E. (1987). Radiation therapy for neoplasms of the brain. J. Neurosurg..

[45] Robin T.P., Rusthoven C.G. (2018). Strategies to preserve cognition in patients with brain metastases: A review. Front. Oncol..

[46] Mehta M.P. (2015). The controversy surrounding the use of whole-brain radiotherapy in brain metastases patients. Neuro Oncol..

[47] Scaringi C., Agolli L., Minniti G. (2018). Technical advances in radiation therapy for brain tumors. Anticancer Res..

[48] Gondi V., Pugh S.L., Tome W.A., Caine C., Corn B., Kanner A., Rowley H., Kundapur V., DeNittis A., Greenspoon J.N. (2014). Preservation of memory with conformal avoidance of the hippocampal neural stem-cell compartment during whole-brain radiotherapy for brain metastases (RTOG 0933): A phase II multi-institutional trial. J. Clin. Oncol..

[49] Oskan F., Ganswindt U., Schwarz S.B., Manapov F., Belka C., Niyazi M. (2014). Hippocampus sparing in whole-brain radiotherapy. A review. Strahlenther. Onkol..

[50] Kim K.S., Wee C.W., Seok J.Y., Hong J.W., Chung J.B., Eom K.Y., Kim J.S., Kim C.Y., Park Y.H., Kim Y.J. (2018). Hippocampus-sparing radiotherapy using volumetric modulated arc therapy (VMAT) to the primary brain tumor: The result of dosimetric study and neurocognitive function assessment. Radiat. Oncol..

[51] Atkins K.M., Pashtan I.M., Bussière M.R., Kang K.H., Niemierko A., Daly J.E., Botticello T.M., Hurd M.C., Chapman P.H., Oh K. (2018). Proton stereotactic radiosurgery for brain metastases: A single-institution analysis of 370 patients. Int. J. Radiat. Oncol. Biol. Phys..

[52] Kazda T., Jancalek R., Pospisil P., Sevela O., Prochazka T., Vrzal M., Burkon P., Slavik M., Hynkova L., Slampa P. (2014). Why and how to spare the hippocampus during brain radiotherapy: The developing role of hippocampal avoidance in cranial radiotherapy. Radiat. Oncol..

[53] Shen C.J., Kummerlowe M.N., Redmond K.J., Rigamonti D., Lim M.K., Kleinberg L.R. (2016). Stereotactic radiosurgery: Treatment of brain metastasis without interruption of systemic therapy. Int. J. Radiat. Oncol. Biol. Phys..

[54] Lehrer E.J., Peterson J., Brown P.D., Sheehan J.P., Quiñones-Hinojosa A., Zaorsky N.G., Trifiletti D.M. (2019). Treatment of brain metastases with stereotactic radiosurgery and immune checkpoint inhibitors: An international meta-analysis of individual patient data. Radiother. Oncol..

[55] Monje M.L., Toda H., Palmer T.D. (2003). Inflammatory blockade restores adult hippocampal neurogenesis. Science.

[56] Chan A.S., Cheung M.C., Law S.C., Chan J.H. (2004). Phase II study of alpha-tocopherol in improving the cognitive function of patients with temporal lobe radionecrosis. Cancer.

[57] Raber J., Villasana L., Rosenberg J., Zou Y., Huang T.T., Fike J.R. (2011). Irradiation enhances hippocampus-dependent cognition in mice deficient in extracellular superoxide dismutase. Hippocampus.

[58] Zou Y., Corniola R., Leu D., Khan A., Sahbaie P., Chakraborti A., Clark D.J., Fike J.R., Huang T.T. (2012). Extracellular superoxide dismutase is important for hippocampal neurogenesis and preservation of cognitive functions after irradiation. Proc. Natl. Acad. Sci. USA.

[59] Benigni A., Cassis P., Remuzzi G. (2010). Angiotensin II revisited: New roles in inflammation, immunology and aging. EMBO Mol. Med..

[60] Wright J.W., Harding J.W. (2004). The brain angiotensin system and extracellular matrix molecules in neural plasticity, learning, and memory. Prog. Neurobiol..

[61] Jenrow K.A., Brown S.L., Liu J., Kolozsvary A., Lapanowski K., Kim J.H. (2010). Ramipril mitigates radiation-induced impairment of neurogenesis in the rat dentate gyrus. Radiat. Oncol..

[62] Lee T.C., Greene-Schloesser D., Payne V., Diz D.I., Hsu F.C., Kooshki M., Mustafa R., Riddle D.R., Zhao W., Chan M.D. (2012). Chronic administration of the angiotensin-converting enzyme inhibitor, ramipril, prevents fractionated whole-brain irradiation-induced perirhinal cortex-dependent cognitive impairment. Radiat. Res..

[63] Robbins M.E., Payne V., Tommasi E., Diz D.I., Hsu F.C., Brown W.R., Wheeler K.T., Olson J., Zhao W. (2009). The AT1 receptor antagonist, L-158,809, prevents or ameliorates fractionated whole-brain irradiation-induced cognitive impairment. Int. J. Radiat. Oncol. Biol. Phys..

[64] Conner K.R., Forbes M.E., Lee W.H., Lee Y.W., Riddle D.R. (2011). AT1 receptor antagonism does not influence early radiation-induced changes in microglial activation or neurogenesis in the normal rat brain. Radiat. Res..

[65] Parihar V.K., Limoli C.L. (2013). Cranial irradiation compromises neuronal architecture in the hippocampus. Proc. Natl. Acad. Sci. USA.

[66] Fidaleo M., Fanelli F., Ceru M.P., Moreno S. (2014). Neuroprotective properties of peroxisome proliferator-activated receptor alpha (PPARα) and its lipid ligands. Curr. Med. Chem..

[67] Ramanan S., Kooshki M., Zhao W., Hsu F.C., Riddle D.R., Robbins M.E. (2009). The PPAR alpha agonist fenofibrate preserves hippocampal neurogenesis and inhibits microglial activation after whole-brain irradiation. Int. J. Radiat. Oncol. Biol. Phys..

[68] Greene-Schloesser D., Payne V., Peiffer A.M., Hsu F.C., Riddle D.R., Zhao W., Chan M.D., Metheny-Barlow L., Robbins M.E. (2014). The peroxisomal proliferator-activated receptor (PPAR) α agonist, fenofibrate, prevents fractionated whole-brain irradiation-induced cognitive impairment. Radiat. Res..

[69] Zhao W., Payne V., Tommasi E., Diz D.I., Hsu F.C., Robbins M.E. (2007). Administration of the peroxisomal proliferator-activated receptor gamma agonist pioglitazone during fractionated brain irradiation prevents radiation-induced cognitive impairment. Int. J. Radiat. Oncol. Biol. Phys..

[70] Reis D.J., Casteen E.J., Ilardi S.S. (2019). The antidepressant impact of minocycline in rodents: A systematic review and meta-analysis. Sci. Rep..

[71] Zhang L., Li K., Sun R., Zhang Y., Ji J., Huang P., Yang H., Tian Y. (2014). Minocycline ameliorates cognitive impairment induced by whole-brain irradiation: An animal study. Radiat. Oncol..

[72] Zhang L., Huang P., Chen H., Tan W., Lu J., Liu W., Wang J., Zhang S., Zhu W., Cao J. (2017). The inhibitory effect of minocycline on radiation-induced neuronal apoptosis via AMPKα1 signaling-mediated autophagy. Sci. Rep..

[73] Wang Y., Zhou K., Li T., Xu Y., Xie C., Sun Y., Zhang Y., Rodriguez J., Blomgren K., Zhu C. (2017). Inhibition of autophagy prevents irradiation-induced neural stem and progenitor cell death in the juvenile mouse brain. Cell Death Dis..

[74] Plangár I., Szabó E.R., Tőkés T., Mán I., Brinyiczki K., Fekete G., Németh I., Ghyczy M., Boros M., Hideghéty K. (2014). Radio-neuroprotective effect of L-alpha-glycerylphosphorylcholine (GPC) in an experimental rat model. J. Neurooncol..

[75] Chen L., Gao X., Zhao S., Hu W., Chen J. (2015). The Small-Molecule TrkB Agonist 7, 8-Dihydroxyflavone Decreases Hippocampal Newborn Neuron Death After Traumatic Brain Injury. J. Neuropathol. Exp. Neurol..

[76] Yang P., Leu D., Ye K., Srinivasan C., Fike J.R., Huang T.T. (2016). Cognitive impairments following cranial irradiation can be mitigated by treatment with a tropomyosin receptor kinase B agonist. Exp. Neurol..

[77] Cosman K.M., Boyle L.L., Porsteinsson A.P. (2007). Memantine in the treatment of mild-to-moderate Alzheimer’s disease. Expert Opin. Pharmacother..

[78] Brown P.D., Pugh S., Laack N.N., Wefel J.S., Khuntia D., Meyers C., Choucair A., Fox S., Suh J.H., Roberge D. (2013). Memantine for the prevention of cognitive dysfunction in patients receiving whole-brain radiotherapy: A randomized, double-blind, placebo-controlled trial. Neuro Oncol..

[79] Acharya M.M., Christie L.A., Lan M.L., Giedzinski E., Fike J.R., Rosi S., Limoli C.L. (2011). Human neural stem cell transplantation ameliorates radiation-induced cognitive dysfunction. Cancer Res..

[80] Acharya M.M., Roa D.E., Bosch O., Lan M.L., Limoli C.L. (2011). Stem cell transplantation strategies for the restoration of cognitive dysfunction caused by cranial radiotherapy. J. Vis. Exp..

[81] Joo K.M., Jin J., Kang B.G., Lee S.J., Kim K.H., Yang H., Lee Y.A., Cho Y.J., Im Y.S., Lee D.S. (2012). Trans-differentiation of neural stem cells: A therapeutic mechanism against the radiation induced brain damage. PLoS ONE.

[82] Baulch J.E., Acharya M.M., Allen B.D., Ru N., Chmielewski N.N., Martirosian V., Giedzinski E., Syage A., Park A.L., Benke S.N. (2016). Cranial grafting of stem cell-derived microvesicles improves cognition and reduces neuropathology in the irradiated brain. Proc. Natl. Acad. Sci. USA.

[83] Piao J., Major T., Auyeung G., Policarpio E., Menon J., Droms L., Gutin P., Uryu K., Tchieu J., Soulet D. (2015). Human embryonic stem cell-derived oligodendrocyte progenitors remyelinate the brain and rescue behavioral deficits following radiation. Cell Stem Cell.

[84] Soria B., Martin-Montalvo A., Aguilera Y., Mellado-Damas N., López-Beas J., Herrera-Herrera I., López E., Barcia J.A., Alvarez-Dolado M., Hmadcha A. (2019). Human mesenchymal stem cells prevent neurological complications of radiotherapy. Front. Cell. Neurosci..

[85] Duncan T., Valenzuela M. (2017). Alzheimer’s disease, dementia, and stem cell therapy. Stem Cell Res. Ther..

[86] Bergami M., Rimondini R., Santi S., Blum R., Götz M., Canossa M. (2008). Deletion of TrkB in adult progenitors alters newborn neuron integration into hippocampal circuits and increases anxiety-like behavior. Proc. Natl. Acad. Sci. USA.

[87] Groves J.O., Leslie I., Huang G.J., McHugh S.B., Taylor A., Mott R., Munafò M., Bannerman D.M., Flint J. (2013). Ablating adult neurogenesis in the rat has no effect on spatial processing: Evidence from a novel pharmacogenetic model. PLoS Genet..

[88] Tofilon P.J., Fike J.R. (2000). The radioresponse of the central nervous system: A dynamic process. Radiat. Res..

[89] Wu P.H., Coultrap S., Pinnix C., Davies K.D., Tailor R., Ang K.K., Browning M.D., Grosshans D.R. (2012). Radiation induces acute alterations in neuronal function. PLoS ONE.

